# Effect of Bioactive Ingredients on Urinary Excretion of Aflatoxin B1 and Ochratoxin A in Rats, as Measured by Liquid Chromatography with Fluorescence Detection

**DOI:** 10.3390/toxins16080363

**Published:** 2024-08-16

**Authors:** Pilar Vila-Donat, Dora Sánchez, Alessandra Cimbalo, Jordi Mañes, Lara Manyes

**Affiliations:** Biotech Agrifood Lab, Faculty of Pharmacy and Food Sciences, University of Valencia, Avda. Vicent Andrés Estellés s/n, 46100 Burjassot, Spain; pilar.vila@uv.es (P.V.-D.); dora.sanchez@espam.edu.ec (D.S.); lara.manyes@uv.es (L.M.)

**Keywords:** feed, mycotoxin excretion, urine, Wistar rats, functional compounds

## Abstract

Aflatoxin B1 (AFB1) and ochratoxin A (OTA) are highly toxic mycotoxins present in food and feed, posing serious health risks to humans and animals. This study aimed to validate an efficient and cost-effective analytical method for quantifying AFB1 and OTA in rat urine using immunoaffinity column extraction and liquid chromatography with fluorescence detection (IAC-LC-FD). Additionally, the study evaluated the effect of incorporating fermented whey and pumpkin into the feed on the urinary excretion of these mycotoxins. The limits of detection and quantification were determined to be 0.1 µg/kg and 0.3 µg/kg, respectively, for both mycotoxins in feed, and 0.2 ng/mL and 0.6 ng/mL, respectively, in urine. The method demonstrated robust recovery rates ranging from 74% to 119% for both AFB1 and OTA in both matrices. In feed samples, the levels of AFB1 and OTA ranged from 4.3 to 5.2 µg/g and from 5.4 to 8.8 µg/g, respectively. This validated method was successfully applied to analyze 116 urine samples from rats collected during the fourth week of an in vivo trial. The results indicated that the addition of fermented whey and pumpkin to the feed influenced mycotoxin excretion in urine, with variations observed based on the sex of the rats, type of mycotoxin, and exposure dosage.

## 1. Introduction

Mycotoxins, produced by various species of fungi, pose significant risks as they commonly contaminate food and feed ingredients. Aflatoxin B1 (AFB1), notably produced by *Aspergillus flavus* and *A. parasiticus*, stands out as the most potent carcinogen, classified as a human carcinogen (Group 1) by the International Agency for Research on Cancer (IARC), responsible for causing hepatocellular carcinomas in humans [1]. Despite stringent regulations, mycotoxin contamination remains a prevalent issue in global trade. According to the Rapid Alert System for Food and Feed (RASFF), aflatoxins (AF) accounted for 95% of notifications between 2011 and 2021 in the European Union, prompting rejections of imported foods at borders. Cereal-based products have been identified as significant contributors to chronic dietary exposure to AFB1 across all age groups [2]. Recently updated European regulations maintain strict maximum levels (ML) for AF in foods, ranging between 0.1 and 12 μg/kg for AFB1 depending on the food product [3].

On the other hand, ochratoxin A (OTA) exhibits nephrotoxic, hepatotoxic, teratogenic, and immunotoxic effects, and recent studies have linked it to neurodegenerative diseases such as Parkinson’s and Alzheimer’s [4]. The main producers of OTA are *Penicillium verrucosum*, *A. ochraceus*, and *A. niger*. OTA has been classified by the IARC as a possible human carcinogen (Group 2B) [5]. Regarding the updated European Regulation, specific ML for OTA have been established, ranging from 0.5 to 80 μg/kg depending on the food product [3].

Moreover, an anticipated 2 °C rise in global temperatures and prolonged drought periods are expected to increase the likelihood of low to moderate AF contamination in European countries [2]. For instance, Topi and colleagues [6] reported the presence of AF and OTA in wheat and corn crops from Albania (southern Europe), with corn samples exceeding European AFB1 ML by 36%, reaching concentrations of up to 3500 µg/kg. Additionally, other authors detected AFs and OTA, among other mycotoxins, in various cereal crops including corn, wheat, and barley. Notably, AFs and OTA were found in limited samples of the cereals analyzed, with the highest concentrations observed in regions experiencing periods of drought in southern Croatia [7]. These findings underscore the critical importance of continuous monitoring of these mycotoxins and the collection of sufficient data for robust toxicological studies, essential for ensuring food security in a world increasingly exposed to climate change.

Regarding metabolism, once ingested, AFB1 is efficiently absorbed in the small intestine and distributed to the liver, where it undergoes first-pass metabolism [2]. Absorbed AFB1 and its metabolites are excreted in the urine, while elimination in the feces is a route for both unabsorbed AFB1 and biliary excretion of the formed metabolites. AFB1 is accumulated in the liver and to a lesser extent in the kidney and is also found in mesenteric venous blood, while OTA is rapidly absorbed after ingestion but is excreted slowly, causing possible accumulation in the body, which is due to binding plasma proteins and a low-rate metabolism. A series of studies on biomarkers of human OTA exposure found that dietary exposure to OTA was reflected in OTA levels in plasma, serum, urine, and breast milk [8].

Individual variations in mycotoxin levels stem from differences in food intake, contamination levels, intestinal absorption, distribution, and excretion. Consequently, there has been a growing focus on studying mycotoxin metabolism and evaluating its presence in biological fluids, as these results provide valuable insights into the actual risks for consumers. Detecting mycotoxin biomarkers in urine offers a direct method to assess exposure compared to the indirect approach of analyzing food [9].

To understand exposure to AFB1 and OTA, analysis of urinary levels of these mycotoxins has been proposed due to their short excretion half-life. Understanding the biotransformation of these mycotoxins would be beneficial for food safety risk assessment but is challenged by difficulties in performing and replicating in vivo experiments, as well as the lack of suitable analytical methods to detect metabolites at trace levels. Furthermore, in vivo studies in Europe are limited, due to their complexity and, most importantly, new regulations in the care and use of live animals for scientific purposes governed by the internationally established principles of the 3Rs (Replacement, Reduction, and Refinement) [10].

The use of liquid chromatography coupled with fluorescence detection (LC-FLD) provides significant advantages for AF and OTA, enabling accurate quantification at very low levels in urine samples. The fluorescence detection technique enhances sensitivity and selectivity by maximizing the signal-to-noise ratio, even in the presence of trace amounts of mycotoxins, which is crucial for assessing exposure levels and potential health risks associated with mycotoxin contamination in food and feed. Additionally, LC-FLD is valued for its user-friendliness and efficiency, offering rapid analysis times and straightforward operation, which further enhances its utility in both research and routine analytical laboratories.

The aim of this study was to design and conduct an in vivo experiment with Wistar rats that ingested feeds naturally contaminated with AFB1 and OTA. The feeds were tested both with and without the addition of bioactive ingredients, specifically pumpkin (P) and fermented whey (FW). The development of robust and efficient analytical methods capable of quantifying mycotoxins at trace levels in urine is crucial for understanding the toxicokinetics of AFB1 and OTA in male and female Wistar rats. Once validated, this method was used to analyze 116 urine samples collected during the fourth week of the in vivo trial. The study also evaluated the impact of adding FW and P to the feed on mycotoxin excretion. These methods will facilitate the quantification of biomarker levels and their correlation with dietary intake, providing insights into long-term exposure. Given the current scarcity of recent in vivo data on mycotoxin excretion profiles, this study aims to enhance our comprehensive understanding of this complex issue.

## 2. Results and Discussion

### 2.1. Sample Extraction Optimization and Clean-Up

Various extraction protocols were optimized to achieve satisfactory recoveries, minimize matrix interferences, and attain the lowest detection limits for this study. Two extraction methods (M1 and M2) were evaluated for extracting mycotoxins from spiked urine samples. Method M2, which omitted the evaporation step compared to M1, was faster and yielded higher recoveries for AFB1 (94 ± 1.1%) and OTA (91 ± 12%), whereas M1 achieved lower recoveries (AFB1: 73 ± 2.6%, OTA: 59 ± 1.0%) (Table 1). However, M1 required a longer extraction time and a larger sample volume (10 mL versus 5 mL for M2). This could pose challenges as rats may not consistently excrete the same volume daily, potentially affecting the adequacy of the sample size for triplicate analysis.

Based on the above, the M2 method was selected for AFB1 and OTA, achieving a good response in the signal of both analytes. Furthermore, with this simultaneous extraction (AFB1 + OTA) using combined immunoaffinity columns (IAC), recoveries were comparable to methods in which IAC was used for AFB1 or OTA individually, such as the work of Du et al. [11] in which AFB1 recoveries were greater than 81% in canine, feline, and porcine urine samples using IAC Aflaprep. Likewise, in the study carried out by Silva et al. [12] OTA recovery in human urine ranged from 90 to 98% using the IAC OchraTest. Similarly, Al Ayoubi et al. [13] indicated a mean OTA recovery of 93% in human urine using IAC Ochraprep. Furthermore, it was proven that the results of purification by IAC are comparable with the recoveries of the biomarkers OTA (96%) and AFB1 (104%) in human urine, performing an extraction using an Oasis HLB Prime cartridge and UPLC- MS/MS analysis [14].

### 2.2. Method Validation for Mycotoxin Analysis in Feed and Urine in LC-FLD

The mycotoxins studied showed good linearity, with regression coefficients greater than 0.999 in feed and urine (Figures S1–S4 in Supplementary Material). Regarding sensitivity in chromatographic procedures, the limit of detection (LOD) is the injected sample that produces a peak with a height at least 2 to 3 times greater than the noise level, while the limit of quantification (LOQ) is the lowest level for which the method is successfully validated. The LOD of both mycotoxins (AFB1 and OTA) in feed and urine was 0.1 ng/g and 0.2 ng/mL, respectively, while the LOQs were 0.3 ng/g and 0.6 ng/mL, respectively (Table 2 and Table 3). Similar results have been obtained by Du et al. [11] in a study of AFB1 in urine from several species, analyzed by LC-FLD in combination with IAC Aflaprep, in which the LOQ for AFB1 was 0.3 ng/mL. However, for OTA, a lower LOD of 0.001 ng/mL was obtained, due to the application of a previous sample extraction procedure with acidified chloroform starting from a much larger amount of urine [15].

The recovery experiments yielded varied results. In the feed, AFB1 recoveries ranged from 74% to 103%, while OTA recoveries ranged from 83% to 93% (Table 2). In urine, AFB1 recoveries ranged from 94% to 107% and OTA recoveries ranged from 85% to 119% (Table 3).

The specificity of the method was evaluated with respect to interferences from endogenous compounds. Therefore, several blank urine samples were analyzed to determine the specificity of the method by looking for interference peaks within 2.5% of the relative retention time of each compound and an S/N ratio of a possible interference peak in the blank sample below the S/N ratio of the analytes in the same elution zone at the LOD level. No interference peak was observed in the blank samples (Figures S5 and S6 in Supplementary Material).

Both methods were successfully validated with satisfactory recoveries, good precision, and excellent linearity, in accordance with the European Commission Decision on the performance of analytical methods, which stipulates that recovery rates should range between 70 and 120% with RSD of <20% [16]. Kosicki et al. [17] highlighted the reliability and validation of a method combining an IAC procedure before LC determination.

### 2.3. Determination of AFB1 and OTA in Feed

The mycotoxin levels were calculated for 12 exposure groups detailed in Table 4 (12 different diets) while the composition of these 12 exposures is detailed in Section 4.3. Generally, the diets administered to the rats consisted of a mixture of uncontaminated wheat flour (control) and different proportions of corn flour contaminated with AFB1 simulating realistic contamination at the laboratory and barley flour contaminated with OTA, with the presence or absence of bioactive ingredients (FW and P) according to the exposure for each group of rats. The results indicated generally higher OTA levels across all groups (5.4 to 8.8 µg/g) compared to AFB1 levels (ranging between 4.3 and 5.2 µg/g). Regarding contaminated feeds, these were prepared by mixing flours (of corn or barley) in which the fungus that produces mycotoxins (AFB1 and OTA, respectively) was previously inoculated to produce the mycotoxin under controlled conditions, so mycotoxin contamination was influenced by various factors including nutrient concentration and changes in weight due to water loss during the baking process [18]. The disparity in mycotoxin concentrations between OTA and AFB1 is attributed to higher natural contamination of cereal by-products by OTA compared to AFB1 [19]. The feed with the highest concentrations of both mycotoxins (AFB1 and OTA) was the AFB1 + OTA + FW + P feed with 5.2 ± 0.9 µg AFB1/g and 8.8 ± 0.4 µg OTA/g. However, the group with OTA + FW + P feed presented the lowest levels of OTA (5.4 µg/g) (Table 4), perhaps because the different nutrient composition influenced the rheological properties of flour doughs and, therefore, the mycotoxin levels.

### 2.4. Body Weight and Feed Intake in Rats

The body weight (bw) of the rats in the fourth week of the in vivo trial (0.311 ± 0.03 kg and 0.203 ± 0.02 kg in males and females, respectively) and data on average feed consumption over 24 h (13.2 ± 7.4 g and 11.37 ± 6.2 g in males and females, respectively) were collected weekly. These data were used to calculate mycotoxin exposure levels based on previously used doses in various toxicity studies: AFB1 at 250 µg/kg bw/day [20] and OTA in rats at 300–500 µg/kg bw/day [21]. The exposure levels to mycotoxins for the different groups of rats in the fourth week of the study were calculated by multiplying the average daily feed intake per rat (g) by the levels of mycotoxins in the feed (μg/g), and then dividing by the body weight (kg) of the rats. The results of mycotoxin exposure are detailed in Table 5.

Male and female rats were exposed to AFB1 at levels ranging from 176 to 276 µg/kg bw/day and 226 to 387 µg/kg bw/day, respectively. OTA exposure levels in males ranged between 197 and 358 µg/kg bw/day, while in females, they ranged from 162 to 462 µg/kg bw/day. Consequently, rats generally had higher exposure to OTA than to AFB1, with female rats generally showing higher exposure levels than males to both mycotoxins. This trend aligns with the higher levels of OTA contamination (5.4–8.8 µg/g) compared to AFB1 (4.3–5.2 µg/g) in the feed, reflecting real-world scenarios where contaminated flour tends to have higher OTA contamination than AFB1. Furthermore, female rats weighed, on average, 35% less than males, yet their intake was only 14% lower (Table 5).

### 2.5. Urine Creatinine Determination

Creatinine levels were determined in all collected urine samples, totaling 116 samples for this study. Two samples were excluded due to low creatinine concentrations falling below the cutoff of 0.10 mg/mL, independent of sex. Additionally, two urine samples could not be collected: one due to a rat not urinating in the metabolic cage and the other due to natural causes unrelated to the exposure. The urinary creatinine levels of the different groups are detailed in Table 6, ranging between 0.11 and 0.72 mg/mL, with mean creatinine levels ranging from 0.18 to 0.40 mg/mL. These levels are consistent with those reported by Kim et al. [22] where urinary creatinine levels in Sprague–Dawley rats varied from 0.2 to 0.8 mg/mL. Following creatinine and mycotoxin determinations, creatinine-adjusted mycotoxin concentrations (ng/mg) were calculated to account for variability in urine dilution, providing a standardized measure and enhancing precision [23].

### 2.6. Determination of Mycotoxins in Urine Samples

Once validated, the M2 extraction method was successfully utilized to analyze 116 urine samples collected from Wistar rats (both male and female) fed AFB1- and OTA-contaminated feed supplemented with bioactive compounds (at 1%) over a 28-day period. In male rats, urinary concentrations of AFB1 ranged from 45 to 173 ng/mg creatinine, while OTA concentrations ranged from 397 to 994 ng/mg creatinine. Female rats exhibited AFB1 concentrations ranging from 85 to 151 ng/mg creatinine and OTA concentrations ranging from 619 to 1405 ng/mg creatinine. The concentrations of both mycotoxins in the urine samples from the 12 different groups of male and female rats are summarized in Table 7. Overall, OTA levels in urine were consistently higher than those of AFB1. This difference can be attributed to the higher concentration of OTA in the feed ingested by the rats compared to AFB1. Additionally, OTA molecules are known to be more stable and soluble, and are primarily excreted through the kidneys, contributing to their higher levels in urine compared to AFB1. In some cases, RSD >20% is due to large interindividual variation in rats, likely due to diversity in the metabolism of these mycotoxins. Scientific evidence indicates that male rats metabolize AFB1 at a rate two to five times higher than female rats [24].

The highest urinary AFB1 levels were found in rats exposed to AFB1 + OTA + FW-feed in both sexes (173 and 151 ng/mg creatinine in males and females, respectively), while the highest urinary levels of OTA were found in rats that ingested OTA- feed (994 and 1405 ng/mg of creatinine in males and females, respectively) followed by those exposed to AFB1 + OTA + FW- feed (811 and 1393 ng/mg of creatinine in males and females, respectively) (Table 7). As expected, no mycotoxins were detected in the urine of the control groups (wheat flour-based feed, feed + FW, and feed FW + P), as shown in Table 7. The enhanced renal excretion of AFB1 and OTA in rats that consumed feeds containing FW may be attributed to the bioactive compounds, particularly lactic acid bacteria (LAB), present in FW. LAB are widely recognized for their probiotic properties and have been extensively studied for their ability to bind to or degrade environmental contaminants such as mycotoxins, toxic metals, and pesticides [18,25]. Experimental in vitro studies have consistently demonstrated that LAB can degrade or reduce the levels of mycotoxins upon exposure. Moreover, in vivo studies on experimental animals have suggested that certain probiotic strains can mitigate intestinal absorption and enhance the excretion of toxic substances from the gastrointestinal tract, suggesting that probiotic cultures could be a promising approach for human body detoxification [26].

### 2.7. Effect of Bioactive Ingredients on Urinary Mycotoxin Excretion

**Figure 1:**
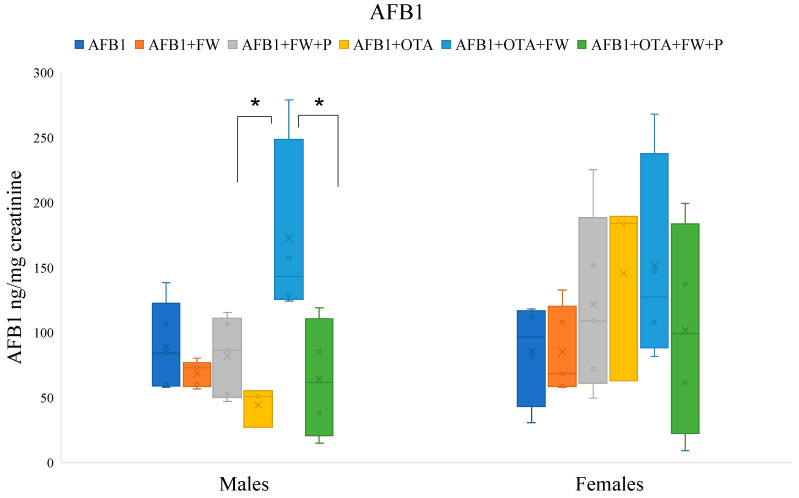
Effects of fermented whey (FW) and pumpkin (P) on urinary aflatoxin B1 (AFB1) levels in male and female Wistar rats. (*) indicates statistically significant differences (*p* ≤ 0.05) in AFB1 urinary levels between experimental groups.

**Figure 2:**
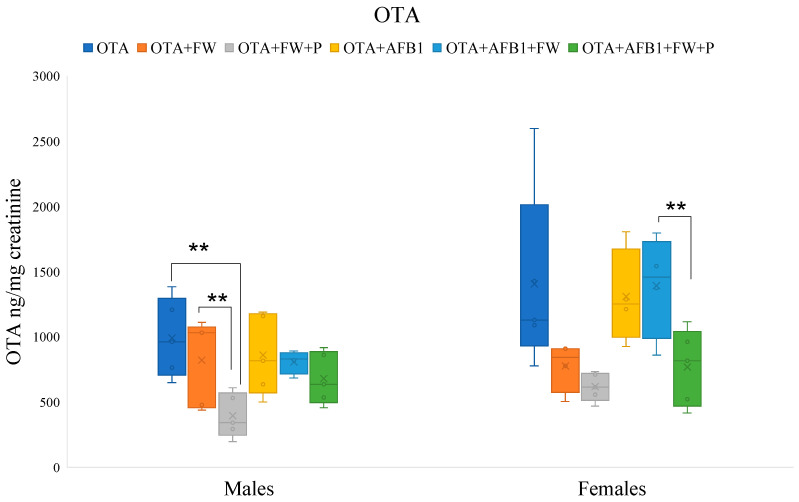
Effects of fermented whey (FW) and pumpkin (P) on ochratoxin A (OTA) urinary levels in male and female rats. (**) indicate statistically significant differences (*p* ≤ 0.01) in OTA urinary levels between experimental groups.

To assess the ability of bioactive ingredients to modify the excretion ratio of AFB1 and OTA in urine, all exposures (excluding the controls free of mycotoxins) were compared. Therefore, Student’s *t*-test was performed to evaluate differences among pairs of conditions containing the mycotoxin (AFB1 or OTA) individually, or a combination of both (AFB1 + OTA), in male and female specimens (Figure 1 and Figure 2).

#### 2.7.1. Effects of Bioactive Compounds on AFB1 Excretion

The urinary AFB1 levels of the group that ingested AFB1 + OTA + FW were significantly higher (*p* ≤ 0.05) than those of the group without FW (AFB1 + OTA) in male rats. There were no significant differences in females although the same trend was observed (Figure 1). On the other hand, the urinary levels of AFB1 in the group that ingested feed AFB1 + OTA + FW + P were significantly lower (*p* ≤0.05) compared to those obtained by group AFB1 + OTA + FW in males, indicating that the combination of FW and P influences urinary AFB1 excretion (Figure 1).

#### 2.7.2. Effects of Bioactive Compounds on OTA Excretion

In male rats, the urinary levels of OTA in the group that ingested the feed (OTA + FW + P) were significantly lower (*p* ≤ 0.01 and *p* ≤ 0.05) than those that ingested the OTA-feed or OTA + FW-feed, respectively. Furthermore, in females, the urinary concentration of OTA in the group (OTA + AFB1 + FW + P) was significantly lower (*p* ≤ 0.05) than in the group that ingested (OTA + AFB1 + FW) (Figure 2). It was observed that FW and P influence OTA urinary excretion depending on the sex of the rat. Previous studies on rats have shown that tissue concentrations of OTA are higher in males than in females, indicating a greater susceptibility of males to OTA toxicity [21].

The different excretion levels of mycotoxins in urine when P is present in supplied feeds could be related to the bioactive compounds present such as vitamin E, which can influence the biotransformation of these mycotoxins. As previously shown by other authors, in the urine of rats fed supplemented diets with vitamin E (600 mg/kg diet), higher levels of unmetabolized AFB1 were found compared to those groups fed without vitamin E or with a lower dose. This indicates that dietary vitamin E could have a significant effect on AFB1 excretion [27]. Furthermore, carotenoids revealed the ability to reduce mycotoxins such as AFB1 in rat tissues [28]. FW and P, being rich in carotenoids (lycopene, α- and β-carotene, lutein, and zeaxanthin), fiber, and biological peptides, play an important role in protecting cells from oxidation and cellular damage, preventing the incidence of human diseases, such as mutagenic processes or cardiovascular diseases [29,30]. These bioactive ingredients (FW and P) added to artisanal contaminated breads showed reduced bioaccessibility of AFB1 and OTA in vitro and may counteract the toxic effects produced by mycotoxins [18,31]. The detoxification potential of whey powder against OTA’s harmful effects was investigated in broilers, showing a significant reduction in the hematobiochemical parameters raised by OTA exposure, as well as a reduction in the OTA residues detected in several organs including the kidney, suggesting the potential application of whey ingredient in broiler feeds to reduce the negative effects of OTA in animals as efficiently as commercial mycotoxin binders [32].

To comprehensively understand the impact of bioactive compounds on mycotoxin biotransformation and excretion, this study could be enhanced by evaluating mycotoxin levels in feces and in target organs, which are the liver and kidneys, of the studied rats. AFB1, for instance, undergoes extensive metabolism primarily in the liver, with some metabolism occurring in the kidneys, while OTA is predominantly metabolized in the kidneys. Additionally, unmetabolized AFB1 is excreted via feces [33]. Monitoring OTA levels in urine can indicate acute exposure, but its long plasma half-life and low urinary excretion rates complicate linking OTA levels in body fluids to daily external intake [34]. This multi-organ and excretion route assessment would provide a more comprehensive understanding of how bioactive compounds influence mycotoxin dynamics in the body.

## 3. Conclusions

A rapid and economical HPLC-FLD method for determining AFB1 and OTA in urine from Wistar rats has been validated and applied to 116 samples from both sexes, collected during an in vivo study. The key advantages of this method include the simultaneous extraction of AFB1 and OTA using a pooled IAC and minimal sample volume requirement (5 mL urine), providing reliable results across a wide concentration range with satisfactory recovery values.

OTA concentrations in rat diets exceeded those of AFB1, resulting in higher exposure levels to OTA and greater urinary excretion of this mycotoxin. Among urine samples, the highest AFB1 concentration was observed in the group fed fermented whey-enriched diets (AFB1 + OTA + FW) in both male and female rats. The addition of bioactive compounds (fermented whey and pumpkin) to the diets influenced mycotoxin excretion in urine, with effects varying depending on rat sex, mycotoxin type (individual or combined), and exposure dosage.

The method’s sensitivity, speed, and versatility suggest its potential application, following further optimization, to other biological fluids and target organs, namely the liver and kidneys. This research contributes with essential recent data on the in vivo toxicokinetics of two significant mycotoxins, addressing a critical gap in current knowledge.

## 4. Materials and Methods

### 4.1. Standards and Solutions

The standards of OTA and AFB1 were purchased from Sigma-Aldrich (St. Louis, MO, USA), with a purity of ≥98% (HPLC). Individual stock solutions of mycotoxins were prepared in methanol (MeOH) at 100 µg/mL, and serial dilutions of these were prepared. All working solutions were protected from light and stored at −20 °C.

### 4.2. Chemical and Reagents

Acetonitrile (ACN) and MeOH, both HPLC/MS grade, were supplied by Fisher Scientific (Loughborough, UK). Ultrapure water (<18.2 MΩ cm resistivity) was obtained in the laboratory using a Milli-QSP^®^ Reagent Water System (Millipore, Beadford, MA, USA). Acetic acid glacial (CH_3_COOH, grade > 99%) was supplied by Fisher Scientific (Loughborough, UK); phosphate-buffered saline (PBS) tablets were provided by Fisher Scientific (Belgium, UK); creatinine 98% was obtained from Acros organics (Loughborough, UK); and picric acid (C_6_H_2_OH(NO_2_)_3_) (98%) from Panreac (Barcelona, Spain) and NaOH came from VWR (Radnor, PA, USA).

### 4.3. Diet, Animals, and Study Design

The animals (120 rats: 60 males and 60 females weighing 260–340 g) were provided by the faculty animal facility (Faculty of Pharmacy and Food Sciences, University of Valencia, Spain). At the beginning of the study, Wistar rats were 4 weeks old. They were housed in polycarbonate cages in a windowless room with a 12 h light–dark cycle. The study room was maintained under controlled conditions appropriate for the species (22 °C, relative humidity 45–65%). To maintain a sterile condition, nitrile gloves and FFP3 masks were used in all procedures performed, including the handling of exposed animals or contaminated samples. The Institutional Animal Care and Use Committee of the University of Valencia (2021/VSC/PEA/0112) approved this project.

Subsequently, the rats were divided into twelve groups based on the administered feed. One control group was fed wheat flour-based feed, two control groups were fed supplemented feed (FW or FW + P), and nine groups were fed feeds with different combinations of contaminated flours (AFB1, OTA), FW, and P. Each group consisted of ten rats (five males and five females to assess sex differences), which were fed the corresponding feed for 28 days, with water provided ad libitum.

The diet administered to the rats was prepared in the laboratory according to the recipe provided in Table 8. The ingredients of the feed varied depending on the type of exposure. Regarding flour contamination, it was performed according to Escrivá et al. [18]. Corn and barley were naturally contaminated with AFB1 and OTA-producing fungi. *A. flavus* ITEM 8111 was acquired from the Agri-Food Microbial Culture Collection of the Institute of Food Sciences and Production (ISPA) Bari, Italy) and *A. steynii* 20,510 was acquired from the Spanish Collection of Type Crops (CECT), Science Park of the University of Valencia (Paterna, Spain). Both fungi were inoculated onto grains (corn and barley) and maintained under optimal laboratory conditions to produce specific mycotoxins (AFB1 or OTA, respectively). To that aim, 300–350 g of maize or barley were introduced in previously autoclaved 1 L glass jars. Then, cereals were contaminated with 15 mL of spores (1 × 10^9^ spores/mL) and mycelium suspension in peptone water with Tween 80 (0.1% both) of the corresponding fungal strain. Glass jars were then left at room temperature in darkness for one month. After that, cereals were autoclaved to remove the fungal contamination, and samples were ground to flour until complete homogenization. Mycotoxins in contaminated flour were quantified by HPLC–MS/qTOF after a solid–liquid extraction, as detailed in Escrivá et al. [18]. Wheat flour, mineral water, salt (NaCl), and sugar (sucrose) were purchased in a supermarket in Valencia (Spain).

Regarding the bioactive ingredients, the whey filtered from goat milk coagulated with commercial rennet (starter culture R-604) was obtained from the company ALCLIPOR, S.A.L. (Benassal, Spain). For whey fermentation, 4 mL of a suspension of lactic acid bacteria (LAB) at a concentration of 10^8^ CFU/mL was added to 40 mL of whey, previously pasteurized according to standardized guidelines, and the samples were incubated (72 h at 37 °C) to allow LAB fermentation. The FW was then lyophilized to obtain a homogeneous powder [18]. The study pumpkin was obtained from a commercial supermarket in Valencia (Spain). Pumpkin powder was prepared by peeling and cutting the fresh vegetable (previously removing the skin and seeds) followed by freeze-drying and grinding to obtain a homogeneous powder. Both ingredients (FW and P) were analyzed to confirm the absence of mycotoxins and stored at −20 °C until use.

For artisanal feed production, a basic recipe to make 1 kg of feed was extrapolated to the initial amount needed for each group (4.6 kg). The preparation of the control feed was carried out using the basic recipe previously described by Lázaro et al. [35] and Escrivá et al. [18], with several modifications: 2800 g of wheat flour, 1727 mL of mineral water, 93 g of sugar (sucrose), and 47 g of salt (NaCl). Contaminated and supplemented feeds were then prepared with slight modifications to the control recipe. Subsequently, for the preparation, a final weight of 3.5 kg was considered, bioactive compounds (FW and P) were included at 1%, and to include the contaminated flours, a fraction of wheat flour was replaced by 467 g of contaminated barley flour or 1381 g of contaminated corn flour (Table 8). After mixing all the ingredients, the doughs were homogenized in a bakery machine (Silver Crest) for 5 min and shaped in pellet form. After that, feeds were covered with silver foil and baked at 200 °C for 45 min in a Memmert ULE 500 muffle furnace (Madrid, Spain). Finally, feeds were cooled at room temperature, obtaining 12 different exposure groups: (1) control feed, (2) AFB1 feed, (3) OTA feed, (4) AFB1 + OTA feed, (5) control feed + FW, (6) FW+ AFB1 feed, (7) FW + OTA feed, (8) FW + AFB1 + OTA feed, (9) control feed FW + P, (10) FW + P+ AFB1 feed, (11) FW + P + OTA feed, and (12) FW + P + AFB1 + OTA feed (Table 8).

The addition of these quantities of contaminated flours aimed to reach final concentrations of 7.0 and 11.4 μg/g for AFB1 and OTA, respectively, to create a realistic mycotoxin ingestion scenario, derived from a biological comparison with the Mediterranean diet and human habits. The differences in the amounts of contaminated flours added (1381 g for AFB1 and 467 g for OTA) were related to the contamination of natural cereals, which is generally higher for OTA compared to AFB1 [19]. Mycotoxins in contaminated flours were quantified using LC-FLD following solid–liquid extraction, as detailed in Section 4.6. Multiple determinations were conducted on these flours to mitigate errors stemming from the heterogeneity of the feeds.

### 4.4. Collection of Urine Samples

**Figure 3:**
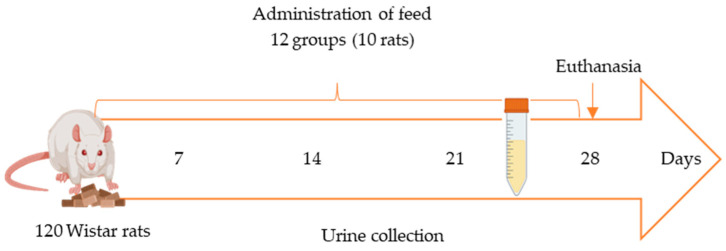
In vivo study scheme using metabolic cages for 24 h once per week starting from second week of exposure.

Fecal and urine samples were collected weekly from each animal (Figure 3). Urine samples (n = 120) were obtained in the fourth week of the in vivo study by individually housing each animal in a metabolic cage for 24 h following the initiation of feed exposure. Within the cage, feces and urine were collected separately in tubes (Figure 3) to ensure the integrity and reliability of the samples. All samples were promptly frozen at −20 °C in Falcon tubes. Urine samples used as blanks for method validation were collected from rats fed the control feed and confirmed to be free of mycotoxins through analysis.

After the 28-day exposure period, the rats were euthanized using isoflurane inhalation, and organs such as the liver and kidneys were stored at −80 °C.

### 4.5. Urine Creatinine Determination

Urinary creatinine levels were determined using the alkaline picrate kinetic method named the Jaffé reaction. The centrifuged urine was diluted (1:5) (4000 rpm for 10 min at 4 °C) and 500 µL was mixed with 1250 µL of milli-Q H_2_O, with the addition of 250 µL of alkaline picrate (0.2 g of picric acid mixed with 250 mL of 1N NaOH). A calibration curve was performed to quantify the samples using increasing concentrations of a 50 µg/mL creatinine standard mixed with milli-Q H_2_O and 250 µL of alkaline picrate. The determination of creatinine consists of the formation of a yellow–orange complex with creatinine and picric acid, whose absorbance is measured at a wavelength of 500 nm [36]. A VWR UV-1600PC spectrophotometer was used for measurement in this study. This determination was performed to adjust for mycotoxin concentrations due to variability in the degree of urine dilution [23].

### 4.6. Mycotoxin Extraction Procedure

#### 4.6.1. Extraction of AFB1 and OTA from Feed

The extraction of AFB1 and OTA from cereal-based feed was carried out by liquid–solid extraction according to Escrivá et al. [18] with some modifications. The feeds were ground in a SHARDOR model CG628B mill, 5 g of the sample was weighed and transferred to a Falcon centrifuge tube (50 mL), and 25 mL of MeOH (80%) was added. Extraction was performed in Ultraturrax (T18 digital ULTRA-TURRAX^®^, Staufen, Germany) for 5 min, then centrifuged at 4000 rpm for 5 min at 4 °C using an Eppendorf 5810R centrifuge (Eppendorf, Hamburg, Germany). Finally, 1 mL of the supernatant was filtered using 0.22 µm nylon syringe filters from Membrane Solutions (Valencia, Spain) into amber vials and injected into HPLC-FLD for AFB1 or OTA determination.

#### 4.6.2. Extraction of AFB1 and OTA from Urine

After creatinine determination, urine samples were thawed and centrifuged at 4000 rpm for 10 min in Falcon tubes. Mycotoxin extraction from urine samples followed methods based on Rubert et al. [37] and Al Ayoubi et al. [13] with modifications. Two similar extraction methods (M1 and M2) were evaluated. Method M1 involved mixing 10 mL of phosphate-buffered saline (PBS) with 10 mL of centrifuged urine and shaking for 3 min. The buffered urine was then concentrated using AflaOchra IAC (Vicam, Watertown, USA), containing antibodies specific for AFB1 and OTA at a flow rate of approximately 1 drop/s, with the eluate discarded. The column was washed with 10 mL of PBS at a flow rate of 1–2 drops/s, and the eluate was discarded. OTA and AFB1 were eluted by slowly passing 5 mL of methanol (MeOH) through the column into a 15 mL Falcon tube. The elution was ensured to be complete by passing air through the column to dryness. Subsequently, the eluate was evaporated to dryness at 50 °C using a nitrogen stream in a Turbovap evaporator and finally reconstituted in 1 mL of 50% MeOH before injection into LC-FLD.

**Figure 4:**
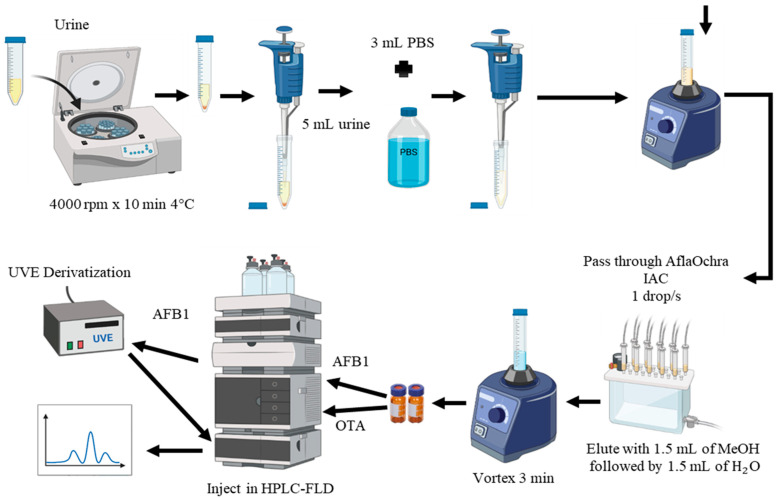
Extraction method M2 of aflatoxin B1 (AFB1) and ochratoxin A (OTA) in urine using AflaOchra immunoaffinity columns (IACs) and liquid chromatography with fluorescence detection (LC-FLD).

Method M2 (Figure 4) was selected for the analysis of urine samples due to its excellent recovery results and rapid extraction (Table 1). In this method, 3 mL of phosphate-buffered saline (PBS) was added to 5 mL of centrifuged urine and shaken for 3 min using a vortex mixer. The buffered urine was then purified using AflaOchra IAC (Vicam, Watertown, USA). The mixture was passed through the IAC column at a flow rate of approximately 1 drop/s, with the eluate discarded. The columns were washed with 5 mL of water (H_2_O) at a flow rate of 1–2 drops/s, and the eluate was discarded. OTA and AFB1 were eluted by slowly passing 3 mL of MeOH–water (1:1) through the column into a glass vial. Elution completeness was ensured by passing air through the columns to dryness. The extracted samples were then transferred to vials and directly injected into the LC-FLD system, as described in the following sections (Figure 4).

### 4.7. Validation Methodology

The analytical methods for AFB1 and OTA were validated based on the following performance characteristics: selectivity, linearity, precision (within- and between-day variability), recovery, LOD, and LOQ. Linearity, sensitivity, and recoveries were assessed for each matrix (feed and urine) in accordance with European Decision 2002/657/EC [17]. Blank samples were obtained from 10 animals (5 males and 5 females) to serve as uncontaminated references.

To evaluate the sensitivity of the method, LOD and LOQ were determined by signal-to-noise ratios (S/Ns) of ≥3 and ≥10, respectively, and were evaluated for both standard solutions and spiked samples. The spiked samples were processed identically to the feeding study samples. The precision of the method was evaluated based on the relative standard deviation (RSD) of repeatability. Intraday precision was evaluated by calculating the RSD from the results generated under conditions of repeatability of six determinations per concentration in a single day. The inter-day precision was calculated by the RSD from the results generated under reproducibility conditions using triplicate determination by concentration over three days.

Linearity was assessed by preparing and analyzing standard calibration curves for each mycotoxin. These curves were used to establish the relationship between the analyte concentration and its response in the measurement system. The calibration points were generated by adding AFB1 or OTA starting from a concentration of 10 µg/mL in MeOH and diluting it with MeOH/H_2_0 1:1 to obtain at least five points (Figure S1, Supplementary Material).

Matrix-matched calibration curves were prepared in the respective matrices (feed and urine) by spiking blank samples with AFB1/OTA standards (10 µg/mL in MeOH) at various concentrations. Calibration points were established to cover a minimum of six levels ranging from 0.02 to 10 µg/g for feed and from 0.25 to 250 ng/mL for urine (Table 2 and Table 3) (Figures S2 and S3, Supplementary Material).

To determine recoveries, feed samples were fortified with AFB1/OTA standards of the known concentration at the beginning of extraction at two levels (1.25 and 2.5 µg/g) and rat urine samples at five levels (0.6, 3.1, 6.25, 12.5, and 50 ng/mL). In the spiked samples, the response was subtracted from the areas obtained in the blank set. Recovery values (%) for all matrices were calculated by dividing the experimental mycotoxin concentration by the theoretical concentration multiplied by 100 (Supplementary Material). All analyses were performed in triplicate. The validation results for the quantitative determination method of AFB1 and OTA (linear regression equation, linearity range, regression coefficients, LOD/LOQ, and recoveries %) of the selected methods for feed and urine are described in Table 2 and Table 3.

AFB1 and OTA measurements were performed by HPLC using an Agilent 1100 series instrument (Agilent Technologies, Santa Clara, CA, USA) equipped with an autosampler, a degasser, a quaternary pump, and an Agilent 1200 FLD detector (Agilent Technologies, Santa Clara, CA, USA), while Agilent software JP03924119 was used for data analysis. A UVE™ photochemical reactor (LCTech, Jasco Analítico S.L, Madrid, Spain) was placed between the analytical column and the FLD detector to enhance the fluorescent activity of AFB1. Chromatographic separation was carried out using a reversed-phase column (150 mm × 4.6 mm, 100 A and 5 µm i.d.) H17-382064 5720-0076 (Phenomenex, Palo Alto, CA, USA). The column temperature was set to 40 °C. For chromatographic analysis of AFB1 in all matrices, the mobile phase used under isocratic conditions consisted of H_2_O/ACN/MeOH (60/10/30 *v*/*v*) with a flow rate of 1 mL/min. The excitation and emission wavelengths were set to 365 and 440 nm, respectively. For chromatographic analysis of OTA, the mobile phase consisted of ACN/H_2_O/CH_3_COOH (55/43/2 *v*/*v*) at a flow rate of 0.8 mL/min in an isocratic regime. The excitation and emission wavelengths for OTA were set to 330 and 460 nm, respectively. The injection volume was 20 and 40 µL for feed and urine samples, respectively. To verify the proper functioning of the instrument and ensure correct parameters, AFB1/OTA standards of known concentrations were injected daily before beginning sample injections.

### 4.8. Statistical Analysis

Statistical analysis of the data (correlation analysis, multiple linear regression analysis, and Student’s *t*-test) was conducted using Microsoft Excel software (2019 version). The differences between the control and exposed groups were analyzed by Student’s *t*-test. The level of *p* ≤ 0.05 was considered statistically significant.

## Figures and Tables

**Table 1:** Optimization of the extraction method of aflatoxin B1 (AFB1) and ochratoxin A (OTA) in spiked urine (methods M1 and M2).

Mycotoxin	Linear Range (ng/mL)	Matrix Calibration Line	$r^{2}$	Recovery (%) 25 ng/mL ± RSD	
				M1	M2
AFB1	0.25–250	y = 0.8647x − 0.2845	0.999	73 ± 2.6	94 ± 1.1
OTA	0.25–250	y = 0.4237x − 0.2551	0.999	59 ± 0.9	91 ± 11.8

LOD, limit of detection. LOQ, limit of quantification. RSD, relative standard deviation.

**Table 2:** Validation results of the LC-FLD method for the analysis of aflatoxin B1 (AFB1) and ochratoxin A (OTA) in feed.

Mycotoxin	Linear Range (µg/g)	Matrix Calibration Curve	$r^{2}$	LOD (ng/g)	LOQ (ng/g)	Recovery (%) ± RSD (%) (*n* = 3)	
						1.25 µg/g	2.5 µg/g
AFB1	0.025–10	y = 0.6265x − 3.9768	0.999	0.1	0.3	74 ± 0.5	103 ± 11
OTA	0.025–10	y = 0.1944x + 0.0214	0.999	0.1	0.3	83 ± 0.4	93 ± 3.3

LOD, limit of detection. LOQ, limit of quantification. RSD, relative standard deviation.

**Table 3:** Validation results of the LC-FLD method for the analysis of mycotoxins in Wistar rat urine (M2).

Mycotoxin	Linear Range (ng/mL)	Matrix Calibration Curve	$r^{2}$	LOD (ng/mL)	LOQ (ng/mL)	Recovery (%) ± RSD (%) (*n* = 3)				
						50 ng/mL	12.5 ng/mL	6.3 ng/mL	3.1 ng/mL	0.6 ng/mL
AFB1	0.2–250	y = 0.8647x − 0.2845	0.999	0.2	0.6	97 ± 5.7	96 ± 12	94 ± 0.1	100 ± 15.5	107 ± 4.6
OTA	0.2–250	y = 4237x + 0.2551	0.999	0.2	0.6	91 ± 13.2	85 ± 0.9	100 ± 1.6	103 ± 12.5	119 ± 8.4

LOD, limit of detection. LOQ, limit of quantification. RSD, relative standard deviation.

**Table 4:** AFB1 and OTA levels in 12 feed groups by LC-FLD analysis (μg/g).

Feed	AFB1 (µg/g)	OTA (µg/g)
Control: wheat flour-based feed	<LOD	<LOD
Feed with AFB1	4.9 ± 0.3	<LOD
Feed with OTA	<LOD	6.0 ± 0.4
Feed with AFB1 + OTA	4.8 ± 0.5	6.4 ± 0.7
Control: Feed + FW	<LOD	<LOD
Feed with AFB1 + FW	4.3 ± 0.2	0.1 ± 0.01
Feed with OTA + FW	0.2 ± 0.0004	8.3 ± 0.1
Feed with AFB1 + OTA + FW	4.5 ± 0.1	7.5 ± 0.2
Control: Feed + FW + P	0.03 ± 0.002	0.06 ± 0.0002
Feed with AFB1 + FW + P	4.7 ± 0.2	0.1 ± 0.001
Feed with OTA + FW + P	<LOD	5.4 ± 0.01
Feed with AFB1+ OTA+ FW + P	5.2 ± 0.9	8.8 ± 0.4

AFB1, aflatoxin B1. FW, Fermented whey. LOD, limit of detection. OTA: ochratoxin A. P: Pumpkin.

**Table 5:** Mycotoxin exposure levels per group of rats (μg mycotoxin/kg body weight).

Group Description	Males		Females	
	AFB1	OTA	AFB1	OTA
	(µg/kg bw/day)			
Control: wheat flour-based feed	-	-	-	-
Feed with AFB1	239	-	309	-
Feed with OTA	-	289	-	417
Feed with AFB1 + OTA	276	312	236	313
Control: Feed with FW	-	-	-	-
Feed with AFB1 + FW	176	-	261	-
Feed with OTA + FW	-	358	-	552
Feed with AFB1 + OTA + FW	189	313	279	462
Control: Feed with FW + P	-	-	-	-
Feed with AFB1 + FW + P	230	-	387	-
Feed with OTA + FW + P	-	197	-	162
Feed with AFB1+ OTA+ FW + P	184	310	226	381

AFB1: Aflatoxin B1; FW: Fermented goat whey; OTA: Ochratoxin A; P: Pumpkin; bw: body weight; (-): no mycotoxin detected. Feed ingestion exposure = (intake of feed (g/day) × levels of mycotoxin in the feed (µg/g))/bw (kg).

**Table 6:** Creatinine levels in urine of Wistar rats (mg/mL) (n = 116).

Group Description	Creatinine Range (mg/mL)	Mean Creatinine (mg/mL)
Control	0.24–0.34	0.29
Feed with AFB1	0.11–0.38	0.27
Feed with OTA	0.11–0.40	0.28
Feed with AFB1 + OTA	0.11–0.35	0.23
Control: Feed + FW	0.30–0.40	0.34
Feed with AFB1 + FW	0.13–0.44	0.28
Feed with OTA + FW	0.16–0.34	0.22
Feed with AFB1 + OTA + FW	0.12–0.25	0.18
Control: Feed + FW + P	0.18–0.22	0.19
Feed with AFB1 + FW + P	0.16–0.41	0.26
Feed with OTA + FW + P	0.23–0.72	0.40
Feed with AFB1+ OTA+ FW + P	0.20–0.66	0.34

AFB1: aflatoxin B1. FW: Fermented whey. OTA: ochratoxin A. P: Pumpkin.

**Table 7:** Concentration of AFB1 and OTA in rat urine samples collected in the fourth week of the in vivo study, normalized by creatinine (ng mycotoxin/mg creatinine) (n = 116).

		Males			Females	
	(ng Mycotoxin/mg Creatinine)					
Group Description	*n*	AFB1	OTA	*n*	AFB1	OTA
Control	5	<LOD	LOD	5	<LOD	<LOD
AFB1	5	89 ± 34	2 ± 4	5	86 ± 40	0.3 ± 1
OTA	5	<LOD	994 ± 304	5	<LOD	1405 ± 706
AFB1 + OTA	5	45 ± 15	862 ± 308	4	145 ± 72	1309 ± 366
Control: FW	5	< LOD	<LOD	5	<LOD	<LOD
AFB1 + FW	5	69 ± 10	3 ± 4	5	85 ± 34	1 ± 2
OTA + FW	5	<LOD	820 ± 331	4	<LOD	777 ± 190
AFB1 + OTA + FW	4	173 ± 73	811 ± 89	4	151 ± 82	1393 ± 395
Control: FW + P	5	<LOD	LOD	5	<LOD	<LOD
AFB1 + FW + P	5	82 ± 31	2 ± 4	5	122 ± 70	0.4 ± 1
OTA + FW + P	5	4 ± 6	397 ± 171	5	3 ± 8	619 ± 109
AFB1+ OTA+ FW + P	5	64 ± 47	683 ± 200	5	102 ± 84	768 ± 294

AFB1: aflatoxin B1. FW: Fermented whey. LOD: limit of detection. OTA: ochratoxin A. P: Pumpkin.

**Table 8:** The amounts (g or mL) of each ingredient used in feed preparation (*n* =12).

Feeds (3.5 kg Final Weight)	Wheat Flour (g)	AFB1- Corn Flour (g)	OTA- Barley Flour (g)	FW (g)	P (g)	Mineral Water (mL)	Sucrose (g)	Salt (g)
Control: wheat flour-based feed	2800	-	-	-	-	1727	93	47
Feed with AFB1	1418	1381	-	-	-			
Feed with OTA	2333	-	467	-	-			
Feed with AFB1 + OTA	952	1381	467	-	-			
Control: Feed + FW	2765	-	-	35	-			
Feed with AFB1 + FW	1384	1381	-	35	-			
Feed with OTA + FW	2298	-	467	35	-			
Feed with AFB1 + OTA + FW	917	1381	467	35	-			
Control: Feed + FW + P	2730	-	-	35	35			
Feed with AFB1 + FW + P	1349	1381	-	35	35			
Feed with OTA + FW + P	2263	-	467	35	35			
Feed with AFB1 + OTA + FW + P	882	1381	467	35	35			

AFB1: aflatoxin B1. FW: Fermented whey. OTA: ochratoxin A. P: Pumpkin.

## Data Availability

The original contributions presented in the study are included in the article material, and further inquiries can be directed to the corresponding authors.

## Supplementary Materials

The following supporting information can be downloaded at: https://www.mdpi.com/article/10.3390/toxins16080363/s1, Figure S1: Standard calibration curve of AFB1 (a) and OTA (b) from 0.2 to 500 µg/L (injection volume = 20 µL); Figure S2: Matrix-matched calibration curve of AFB1 (a) and OTA (b) in spiked feed from 0.1 to 2000 µg/L (injection volume = 20 µL); Figure S3: Matrix-matched calibration curve of AFB1 (a) and OTA (b) in spiked urine from 0.2 to 100 ng/mL (injection volume = 40 µL); Figure S4: Comparison of standard curve and matrix-matched curve for AFB1 (a) and OTA (b) in spiked urine from 0.2 to 500 µg/L to confirm absence of matrix effect (Injection Volume = 20) µL); Figure S5: LC-FLD Chromatograms of AFB1 (a) and OTA (b) in rat urine spiked at 50 ng/mL; Figure S6: LC-FLD chromatogram examples of rat urine samples contaminated with AFB1 (a) and OTA (b) post dietary exposure.
