# Supplementary material for: Effect of Bioactive Ingredients on Urinary Excretion of Aflatoxin B1 and Ochratoxin A in Rats, as Measured by Liquid Chromatography with Fluorescence Detection
Source: Toxins (Basel). 2024 Aug 16;16(8):363. doi: 10.3390/toxins16080363 (PMC11359235; doi:10.3390/toxins16080363)
Supplement: Supplementary file 1 [file toxins-16-00363-s001.zip › toxins-3097838-supplementary.pdf]

Supplementary Materials: Effect of Bioactive Ingredients on Urinary Excretion of Aflatoxin B1 and Ochratoxin A in Rats, as Measured by Liquid Chromatography with Fluorescence Detection

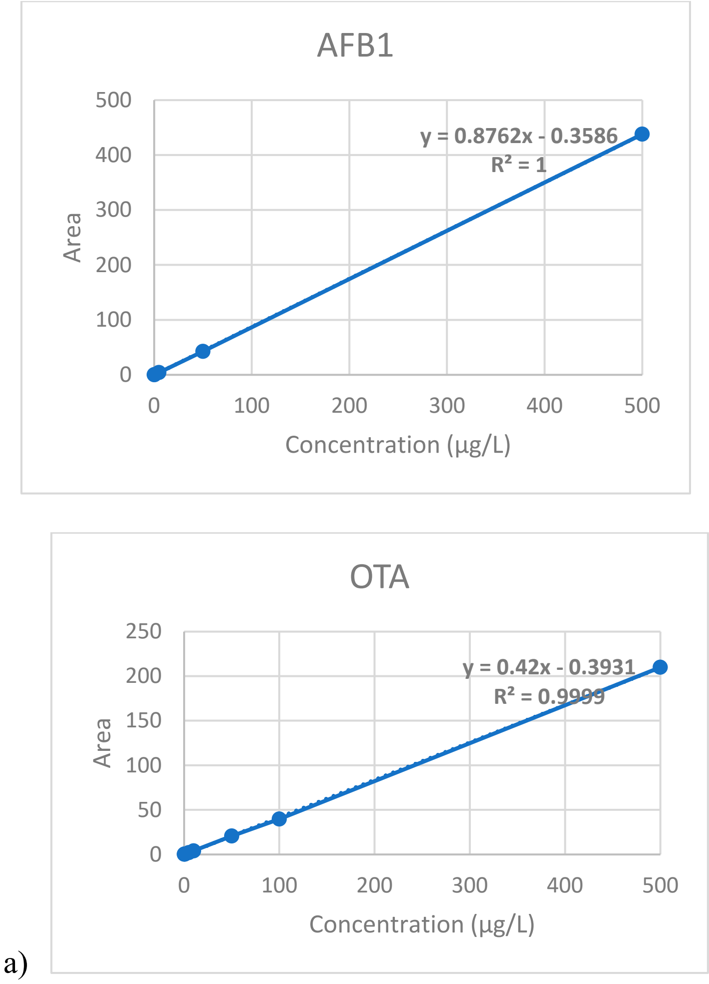

a)

Figure S1. Standard Calibration Curve of AFB1 (a) and OTA (b) from 0.2 to 500 µg/L (injection volume = 20 µL).

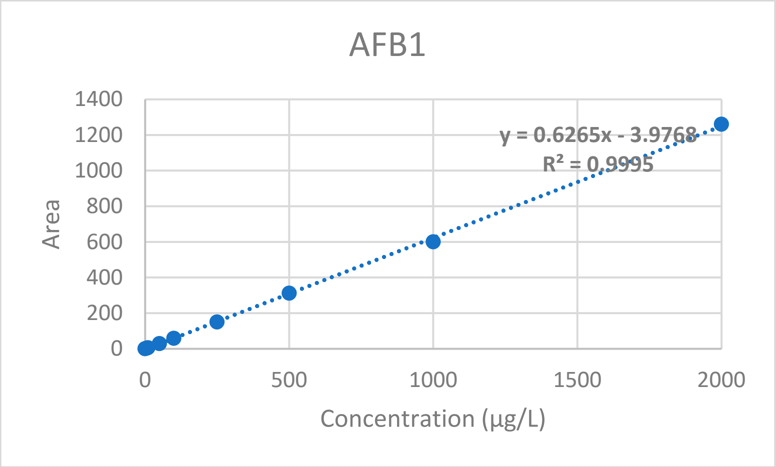

a)

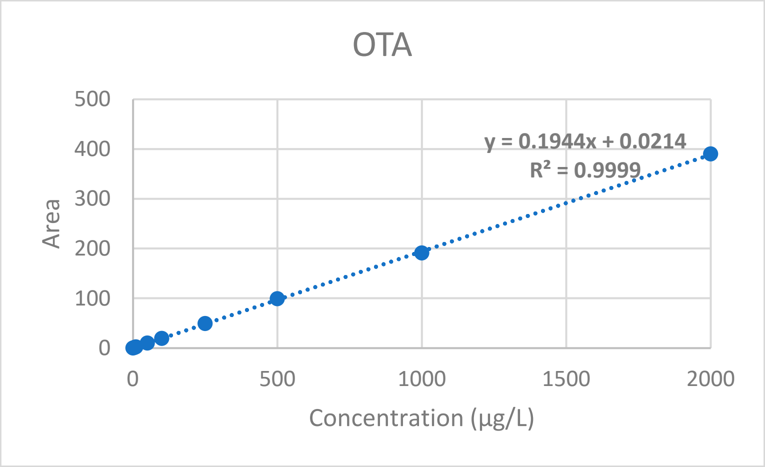

b)

Figure S2. Matrix-Matched Calibration Curve of AFB1 (a) and OTA (b) in spiked feed from 0.1 to 2000 µg/L (injection volume = 20 µL).

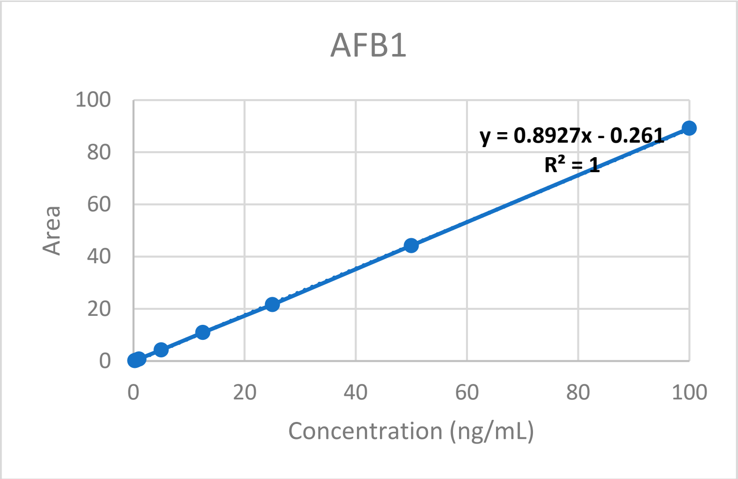

a)

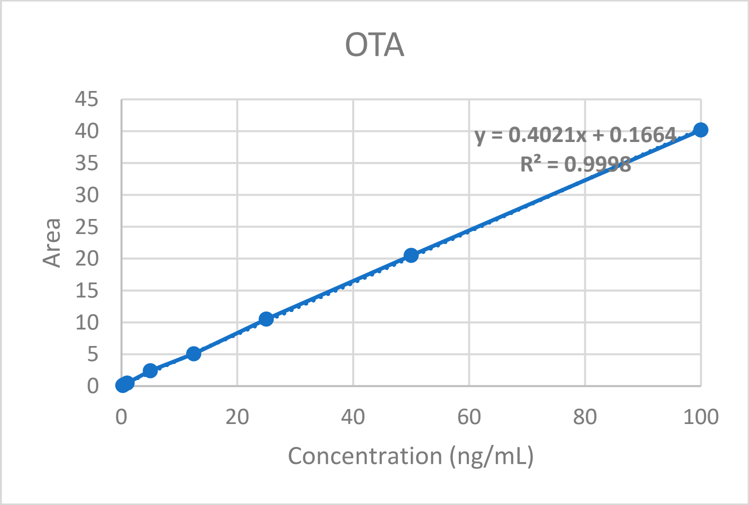

b)

Figure S3. Matrix-Matched Calibration Curve of AFB1 (a) and OTA (b) in spiked urine from 0.2 to 100 ng/mL (injection volume = 40 µL)

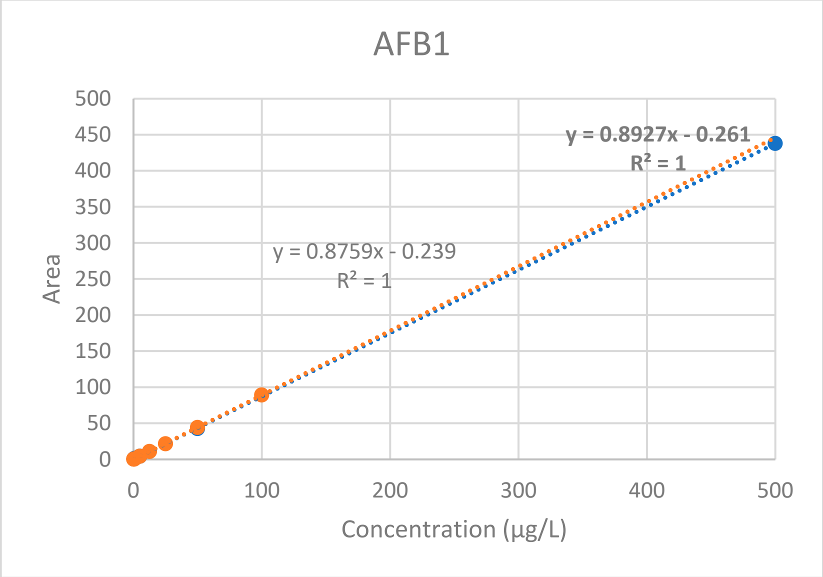

a)

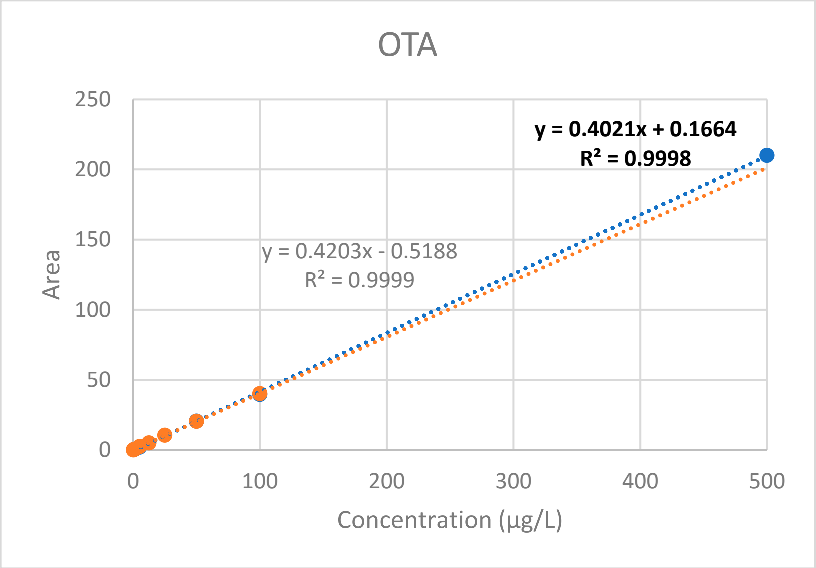

b)

Figure S4. Comparison of Standard Curve and Matrix-Matched Curve for AFB1 (a) and OTA (b) in spiked urine from 0.2 to 500 µg/L to confirm absence of matrix effect (Injection Volume = 20) µL).

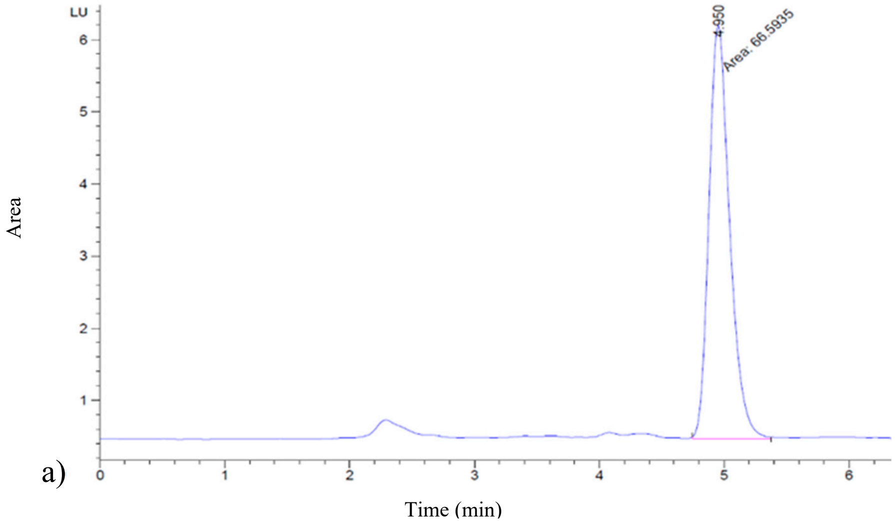

a)

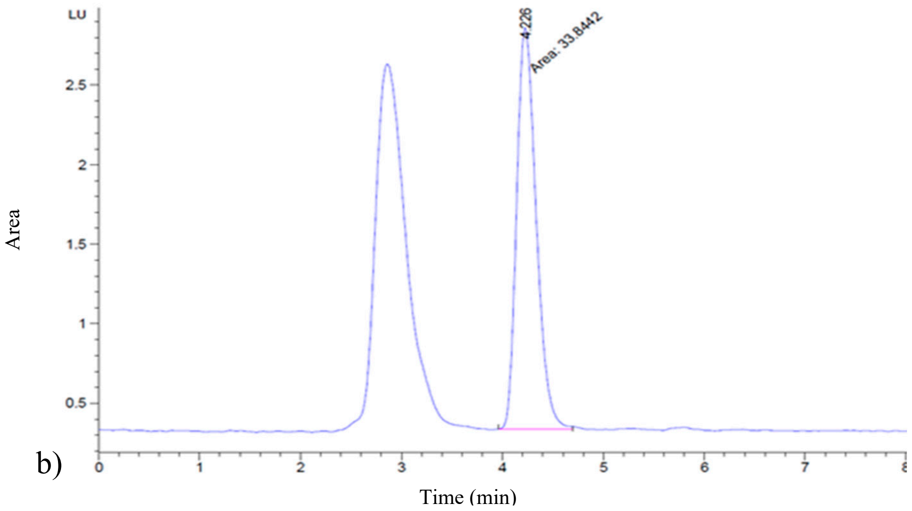

Figure S5. LC-FLD Chromatograms of AFB1 (a) and OTA (b) in rat urine spiked at 50 ng/mL.

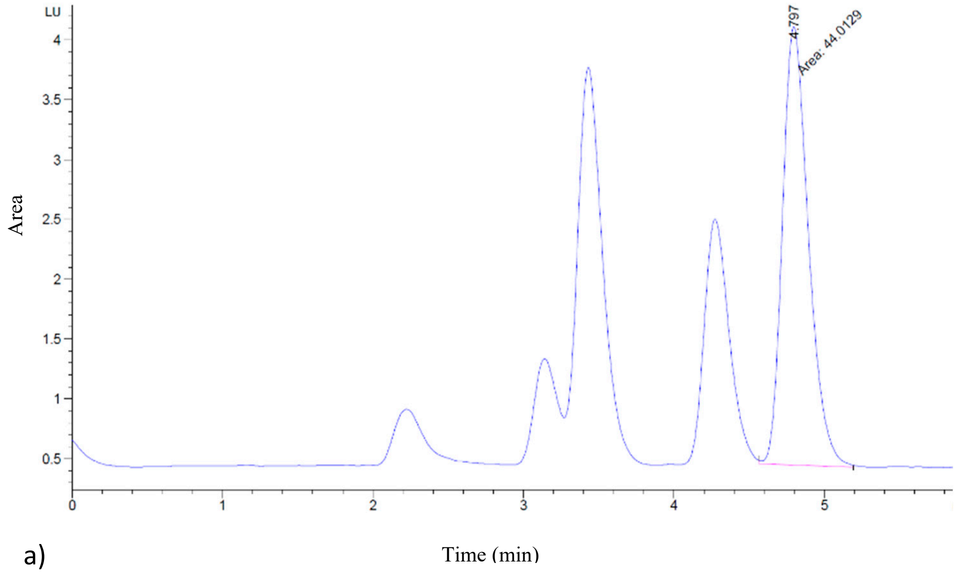

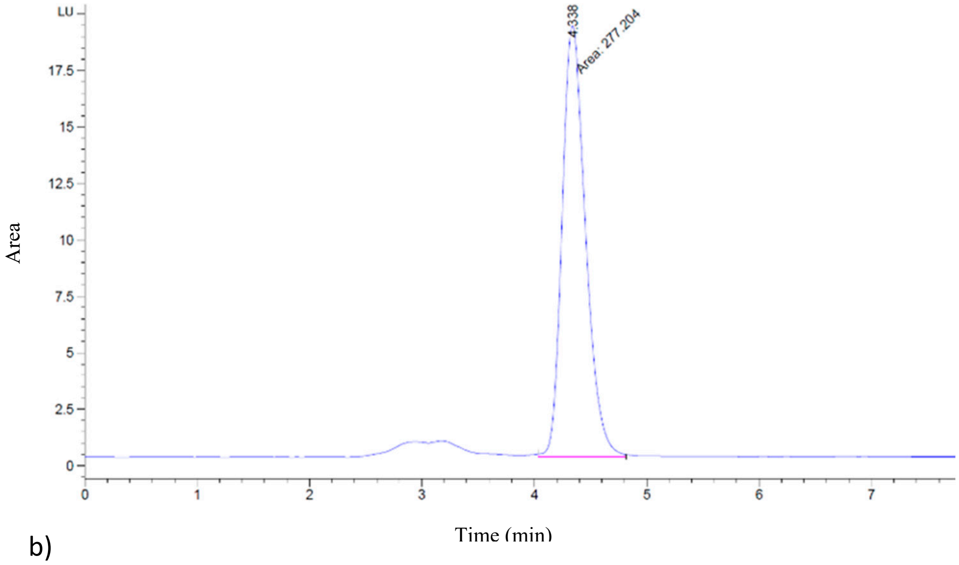

b)

Figure S6. LC-FLD chromatogram examples of rat urine samples contaminated with AFB1 (a) and OTA (b) post dietary exposure.
